# Higher Atherogenic Index of Plasma Is Associated with Hyperuricemia: A National Longitudinal Study

**DOI:** 10.1155/2024/4002839

**Published:** 2024-02-19

**Authors:** Feifei Xu, Chengyong Ma, Shouping Wang, Qin Li, Zhongwei Zhang, Min He

**Affiliations:** ^1^Department of Intensive Care Medicine, West China Hospital, Sichuan University, Chengdu, China; ^2^Department of Critical Care Medicine, West China Hospital, Sichuan University, No. 37, Guoxue Alley, Chengdu 610041, China

## Abstract

**Background:**

The association between atherogenic index of plasma (AIP) and hyperuricemia remains indistinct. This study was aimed to examine the relationship between AIP and hyperuricemia among the middle-aged and the elderly Chinese population.

**Methods:**

Datasets were retrieved from the China Health and Retirement Longitudinal Study (CHARLS) survey conducted in 2011 and 2015. 13,021 participants in the CHARLS in 2011 and 7,017 participants involved both in 2011 and 2015 were included, respectively. The measurement of AIP and hyperuricemia was based on the test of fasting blood. Association between AIP and hyperuricemia was assessed by logistic regression, and the nonlinear association was examined by restricted cubic splines (RCS). The cutoff point of AIP was calculated using receiver operator curve (ROC). 1 : 1 propensity score matching (PSM) was adopted to further explore the relationship between AIP and hyperuricemia.

**Results:**

In the section of a cross-sectional study, a positive association between AIP and hyperuricemia was found. The odds ratios (ORs) of hyperuricemia were 1.00 (reference), 1.52 (1.10–2.10), 1.80 (1.31–2.47), and 3.81 (2.84–5.11). Nonlinear association was not detected using RCS analysis. There were 664 hyperuricemia cases during the four years follow-up. The hyperuricemia prevalence was 9.5%. In the fully adjusted longitudinal analysis, the ORs for hyperuricemia across the quartiles of AIP were 1.00 (reference), 1.00 (0.74–1.37), 1.59 (1.20–2.11), and 2.55 (1.94–3.35), respectively. In the longitudinal analysis after PSM, the OR of hyperuricemia were 1.91 (1.45, 2.51) and 1.92 (1.45, 2.54) in the univariate and multivariate model, respectively.

**Conclusion:**

AIP can predict the prevalence of hyperuricemia in the Chinese middle-aged and elderly population.

## 1. Introduction

Hyperuricemia is a disease defined as an abnormal elevating status of plasma uric acid, which is the final enzymatic product of purine metabolism [1, 2]. The main manifestation of hyperuricemia is gout, which can cause pathological pain in soft tissues or joints, and even result in renal dysfunction at the end stage [1]. Nowadays, hyperuricemia has become a major public health problem that threatens human health, and its prevalence keeps rising constantly [3]. It has been reported that the incidence rate of hyperuricemia in the US was up to 21.2% in male and 21.6% in female [4]. Meanwhile, with the economic development in China, an increasing trend of hyperuricemia and gout has been observed. Literature documented that the overall hyperuricemia prevalence in China was about 13.3%, and the specific morbidity ranges from 10.1% in northeast China to 18.6% in south China due to the demographic constitution and imbalanced economic development [5]. The rapid growth of hyperuricemia impaired the population health, and at the same time, it added the burden of the medical and healthcare system [6]. Moreover, hyperuricemia was demonstrated closely correlated with some critical illness such as cardiovascular disease in recent years [7, 8]. A study from Korea reported hyperuricemia was associated with a nearly tripled risk for heart rate irregularity, which reflecting total arrhythmia [9]. The risk and all-cause mortality of coronary heart disease rises when participants suffer from hyperuricemia [10]. Besides, the high uric acid level can be applied to predict the severity of coronary artery disease [11]. In addition, hyperuricemia also has relationship with a significant increased risk of hypertension and congestive heart failure [12–14]. Thus, early prediction of hyperuricemia is worthy to be explored.

Besides, hyperuricemia has been confirmed to have an association with dyslipidemia, which is a cluster of blood-lipid disorder diseases. Similarly, dyslipidemia is also an independent risk factor for cardiovascular disease [15]. Previous study testified hypercholesterolemia is one of the principal driving forces in the development of atherosclerotic cardiovascular diseases [16]. A large amount of evidence from genetic, epidemiologic, and clinical intervention studies has already firmed that high serum levels of low-density lipoprotein cholesterol cause the development of atherosclerotic plaque [17]. Except that, hyperuricemia is often accompanied by abnormal lipid metabolism, including abnormal apolipoprotein A and low-density lipoprotein cholesterol levels; furthermore, the ratio of apolipoprotein B to A was found strongly associated with serum uric acid levels in US people [18]. AIP, which is calculated as log (triglyceride/high-density lipoprotein), is an indicator for the process of lipid metabolism. It was proposed for the first time by Dobiásová and has close correlation with lipoprotein particle size and esterification rate in apoB-lipoprotein-depleted plasma [19]. Besides, it was also identified as a novel indicator of cardiovascular disease among different populations [20]. As an independent risk factor, AIP is useful for improving the diagnosis and prevention of subclinical coronary artery disease [21]. A low level of AIP can even predict a decrease in all-cause mortality of hospitalized patients with acute myocardial infarction [22], while the relationship between hyperuricemia and AIP has been rarely studied.

Given that uric acid has a close relationship with dyslipidemia, the association between the metabolism indicator AIP and hyperuricemia was examined in this article. To our knowledge, this was the first research to identify the effect of AIP on the occurrence of hyperuricemia in Chinese middle-aged and elderly people on a national scale.

## 2. Methods

### 2.1. Data Source and Analytical Sample

In this paper, a dataset was retrieved from the CHARLS 2011 and 2015 surveys. CHARLS is a longitudinal project initiated from 2011 and followed up every 2 years in China. It is conducted by the Chinese Center for Disease Control and Prevention (CCDCP). The project mainly involves home interviews, medical examination, etc., which were collected from 28 provinces and 150 counties in the whole of China. Information such as health information, demographics, socioeconomic status, as well as physical and physiological measurements was recorded. Specific descriptions regarding the CHARLS could be accessed via the official website (https://charls.pku.edu.cn/) or publications [23].

**Figure 1:**
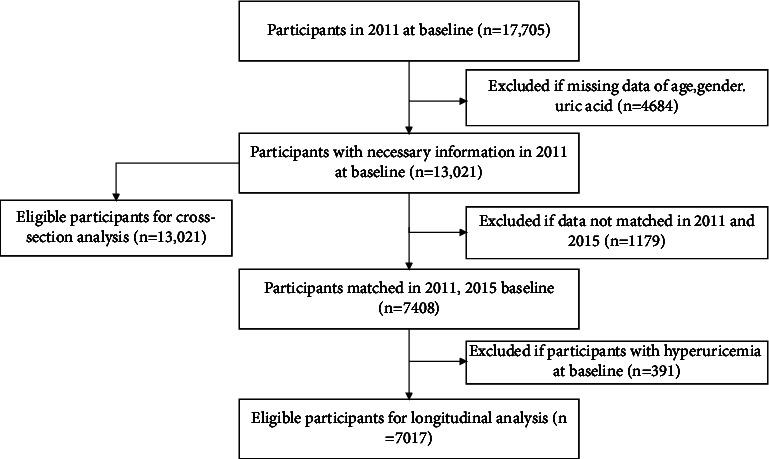
Flowchart of the study population.

A total of 17,705 participants were enrolled in the 2011 baseline. After eliminating the missing data such as age, gender, or uric acid testing, 13,021 eligible participants were reserved for a cross-sectional study. Respondents who participated both in the CHARLS 2011 and 2015 waves were selected from CHARLS 2015. Besides, those with hyperuricemia at baseline in 2011 were excluded. At last, 7,017 participants were reserved for longitudinal analysis. The detail is shown in Figure 1.

### 2.2. Data Collection and Covariates

In the CHARLS survey, questionnaires containing basic information such as marital status, behavior, and history of diseases were handed out by experienced interviewers [23]. Fasting venous blood from the respondents was acquired and centrifuged to gain plasma. The plasma was then immediately stored under appropriate frozen conditions (−20°C) until transporting to the CCDCP in Beijing for testing within 2 weeks. The medical laboratory results were obtained by testing these specimens. Indicators including uric acid, triglyceride (TG), high-density lipoprotein (HDL), blood urea nitrogen (BUN), cystatin (Cyc) C, glucose, and low-density lipoprotein (LDL) were determined using enzymatic colorimetric tests [23]. The covariates in the present study consisted of age, gender, marital status, sleep duration, afternoon napping, cigarette consumption, alcohol consumption, body mass index (BMI), depression reflected by the Center for Epidemiologic Studies Depression (CESD) scale-10 questionnaire, BUN, LDL, hepatic disease, renal disease, digestive disease, and arthritis disease. Age was categorized into 4 groups: 40–50 years, 50–60 years, 60–70 years, and >70 years; marital status was categorized into individuals living together or living alone. Sleeping time was classified into three groups: sleeping less than 6 hours, between 6 and 8 hours, and more than 8 hours. Afternoon napping was defined according to whether participants had the custom of afternoon napping or not. Smoking status consisted of three statuses: currently smoking, never smoking, and quit smoking. Alcohol consumption was categorized as drinking more than once a month, less than once a month, and never drinking. BMI was divided into three groups: <18.5 kg/m^2^, <24 kg/m^2^, <28 kg/m^2^, and ≥28 kg/m^2^. Depression was assessed by CESD-10, and participants got score >10 were identified as depressive state [24]. LDL is classified into two groups >120 mg/dl and <120 mg/dl. Hepatic disorders included hepatitis, liver cyst, and hepatic aneurysm except for fatty liver, tumors, and cancer. Renal disorders included kidney stones and CKDs excluding tumor or cancer. Digestive diseases covered digestive ulcer, gastritis, etc., but did not contain tumor or cancer.

### 2.3. Acquisition of AIP and the Definition of Hyperuricemia

TG and HDL of fasting blood plasma were examined as described above. Then, AIP was calculated using the following formula [19]: AIP = log (TG(mg/dL)/HDL(mg/dL)); participants were classified into four groups according AIP quartiles (*Q*): *Q*1 (−2.83, 0.29), *Q*2 (0.29, 0.77), *Q*3 (0.77, 1.30), and *Q*4 (1.30, 5.33). According to the consensus of multidisciplinary experts on diagnosis and treatment of hyperuricemia-related diseases in China [25], the level of serum uric acid (SUA) to define hyperuricemia was SUA ≥357 *μ*mol/L (6 mg/dL) for females and ≥417 *μ*mol/L (7 mg/dL) for males, which is the dominant definition of hyperuricemia in China [26–28].

### 2.4. Statistical Analyses

Data analysis was conducted using Stata 16.0. Continuous data are presented as the mean ± standard deviation (s.d.), and categorical data are displayed as proportions (%). Analysis of variance was used to evaluate the differences in baseline characteristics, and Student's *t*-test and the Chi-square were adopted for continuous data and categorical data, respectively. Logistic regression was adopted to assess the ORs for the association between AIP and hyperuricemia. The established regression models were displayed as follows. Model 1: univariate regression; Model 2: adjusted by age, gender, marital status, sleeping time and afternoon napping based on model 1; Model 3: further adjusted by alcohol and cigarette consumption, BMI and depression; Model 4: further adjusted by BUN and LDL; Model 5: further adjusted by hepatic disease, digestive disease, renal disease and arthritis disease. Besides, subgroup analysis was employed to explore the association in particular groups. A restricted cubic spline was adopted to model and visualize the non-linear relationship between AIP and hyperuricemia index with 3 knots. To eliminate the possible influence of potential confounders, a 1 : 1 propensity score matching (PSM) without replacement was performed based on the cutoff point calculated from the ROC analysis. In the current investigation, 0.03 was selected as the caliper width for PSM. In the PSM cohort, every possible confounder was considered [29]. Longitudinal analysis was further used to access the association between AIP and hyperuricemia. A sensitive analysis was carried out using the full adjusted Model 5, only within the participants without use of lipid-lowering drugs. Figures were drawn by Stata and GraphPad Prism 8.0 (GraphPad Software Inc., San Diego, CA). *P* < 0.05 (two-sided) was considered statistically significant.

## 3. Results

### 3.1. Demographic Characteristics of the Study Population

A total of 13,021 participants at CHARLS 2011 baseline were included in the cross-sectional analyses. Among them, 6,146 were female and 5,367 were male. Across the quartiles, the mean (SD) of AIP is −0.07 ± 0.28, 0.54 ± 0.13, 1.02 ± 0.15, and 1.87 ± 0.54, respectively. The mean age of the enrolled participants was 59.21 ± 9.71, and the age showed decreased trend across AIP quartiles.  Table 1 shows that the number of individuals whose BMI <24 kg/m^2^ apparently decreased across the quartiles of AIP, but the number increased across the quartiles in the participants with BMI ≥24 kg/m^2^. The prevalence of currents moking was also lower among those with high AIP. It is less likely to have higher AIP in the participants without napping and with digestive disease. The detail of baseline clinical profiles attending the 2011 CHARLS survey is shown in Table 1.

### 3.2. The Association between AIP Index and Hyperuricemia

In the 2011 survey, the prevalence of hyperuricemia in the middle-aged and above Chinese was 5.20%. As shown in Table 2, the prevalence of hyperuricemia was 3.26%, 4.56%, 4.96%, and 10.55% in the four groups, respectively, which indicated an increasing trend across the quartiles of AIP. In Model 1, the ORs of univariate regression at *Q*2, *Q*3, and *Q*4 were 1.42 (1.08–1.86), 1.55 (1.19–2.02), and 3.50 (2.76–4.44); all *p* values have a statistical significance. In the other multivariate regression models, the ORs displayed the same elevating tendency as Model 1. Especially, all *p* values were less than 0.01 in Model 5. In this full adjusted model, the ORs were 1.00 (reference), 1.52 (1.10–2.10), 1.80 (1.31–2.47), and 3.81 (2.84–5.11). The risk of hyperuricemia increased by 250%, 318%, 245%, 281%, and 281% at *Q*4 in the five models, respectively. All *p* values of tendency test were less than 0.001, representing that the risk of hyperuricemia rising with the increasing AIP.

### 3.3. The Subgroup Analysis and Interaction Effect Analysis

Subgroup analysis was employed to further explore the association between AIP and hyperuricemia. The increasing trend still existed in the subgroups of participants without renal disease and sleeping less than 6 hours. Subgroup difference was not observed among gender, renal disease, cigarette consumption, depression, and sleep duration, while drinking more than once a month was detected to exacerbate the association between AIP and hyperuricemia (*P* *for* *interaction*=0.02). Besides, in these subgroups, all ORs at *Q*3 and *Q*4 generally exhibit an increasing trend. Details are exhibited in Table 3.

### 3.4. Results of RCS Analysis, ROC, and Logistic Regression after PSM

**Figure 2:**
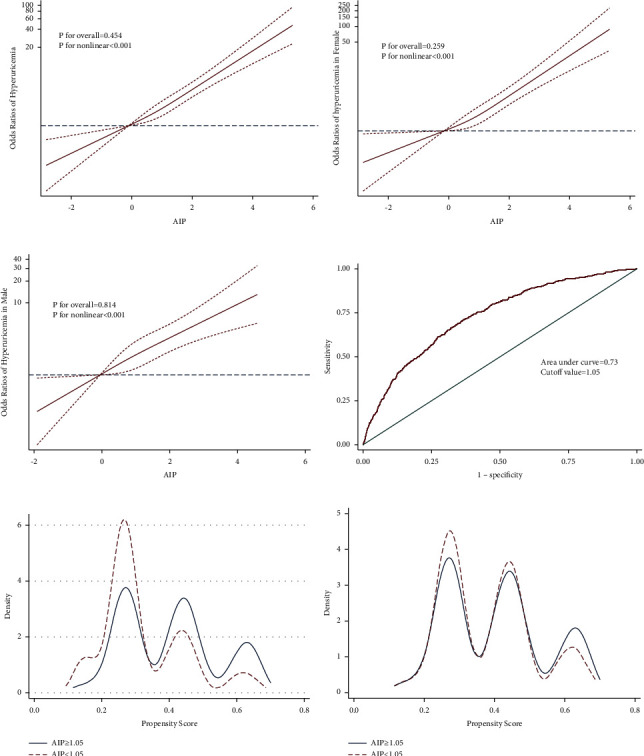
Results of RCS analysis, ROC, and PSM. (a) RCS results for the prevalent hyperuricemia. (b) RCS results for the prevalent hyperuricemia in female. (c) RCS results for the prevalent hyperuricemia in male. The RCS regression model was adjusted as Model 5. RCS: restricted cubic spline. (d) Receiver operator curve. (e) Kernel density plot before PSM. (f) Kernel density plot after PSM.

**Figure 3:**
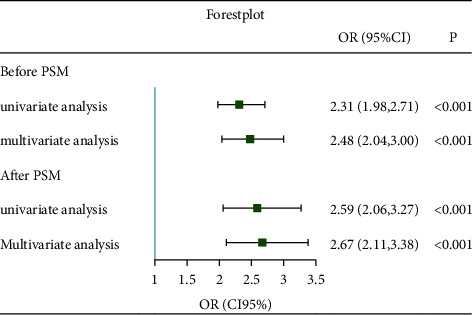
Forest plot of logistic regression before and after PSM. The full adjusted model as Model 5 was used in multivariate logistic regression.

Restricted cubic splines analysis was employed to flexibly model and visualize the association between AIP and hyperuricemia. As shown in Figures 2(a)–2(c), though there was no nonlinear relationship detected, all curves showed elevated trend of ORs with AIP increasing. P for nonlinearity was 0.45, 0.26, and 0.81, respectively, in total, male and female groups. P values for overall were all less than 0.001. ROC displayed that the area under the curve was 0.73, indicating a good predictive power; and the cutoff point of AIP was 1.05 (Figure 2(d)). Figures 2(e) and 2(f) exhibited the Kernel density plot before and after PSM; outcomes showed that distribution of patients' propensity scores more concentrated after PSM, representing the AIP <1.05 group and AIP ≥1.05 group were well matched. After PSM, 2900 pairs of participants were reserved for further logistic regression. The ORs were 2.59 (2.06, 3.27) and 2.67 (2.11, 3.38) in the univariate and multivariate logistic regression after PSM (Figure 3). The baseline profiles after PSM are shown in Supplementary Table 1 and Supplementary Figure 1.

### 3.5. The Longitudinal Association between AIP and Hyperuricemia

In this section, longitudinal analysis was performed in participants involved in both CHARLS 2011 and 2015 survey. Result showed that the overall prevalence of hyperuricemia in target population in 2015 was 9.5%. Across the quartiles of AIP, the incidence gradually increased and was 5.92%, 6.48%, 10.37%, and 15.48%. Besides, the increasing trend still exists in the ORs across the AIP quartiles in every model, and all *p* values for trend were less than 0.001 (Table 4).

Significant subgroup effects on the association between AIP and hyperuricemia was not found in gender, renal disease, alcohol consumption, depression, and sleep duration. Among participants who never smoked, higher AIP was apparently associated with an increased risk of hyperuricemia; the OR was 1.78 (1.22–2.60) and 2.74 (1.90–3.95) compared with the first quartile of AIP (Table 5).

**Figure 4:**
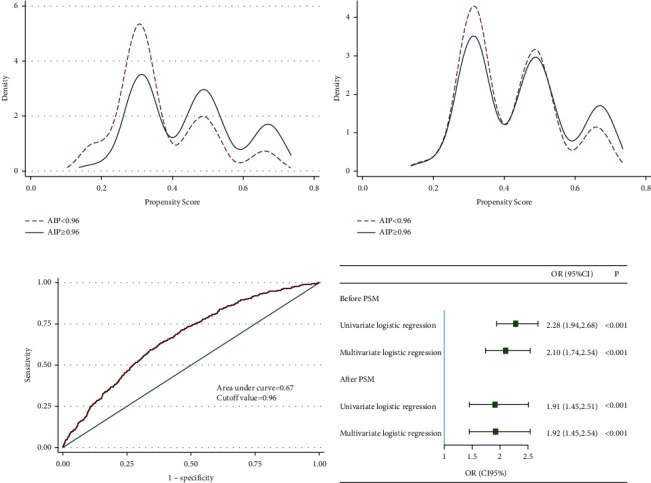
Longitudinal analysis after PSM. (a) Kernel density plot before PSM. (b) Kernel density plot after PSM rather than ROC curve. (c) Receiver operator curve. (d) Forest plot of logistic regression before and after PSM.

PSM was used to reduce the interference of confounding factors. Figures 4(a) and 4(b) show that participants grouped by the cutoff point of 0.96 well matched. The cutoff point was calculated from ROC, and the area under the curve was 0.67 (Figure 4(c)). After PSM, 2096 pairs of participants were reserved for further logistic regression. In longitudinal analysis, the ORs before PSM were 2.28 (1.94, 2.68) and 2.10 (1.74, 2.54) in univariate and multivariate logistic regression before PSM. The baseline profiles after PSM are shown in Supplementary Table 2 and Supplementary Figure 2. After PSM, the ORs were 1.91 (1.45, 2.51) and 1.92 (1.45, 2.54), respectively (Figure 4(d)).

### 3.6. Sensitive Analysis of Treatment for Dyslipidemia

**Figure 5:**
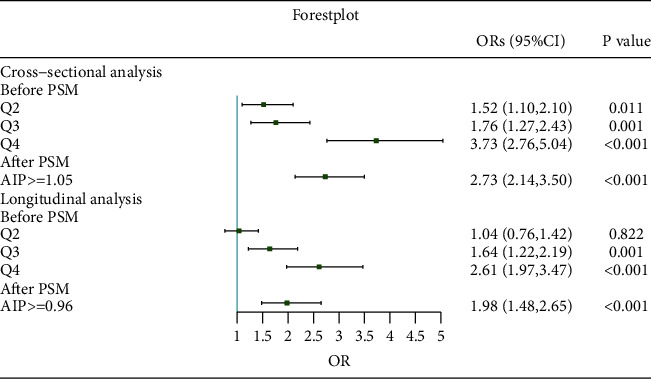
The sensitive analysis of treatment for dyslipidemia AIP: atherogenic index of plasma; OR: odds ratio; PSM: propensity score matching; *Q*: quartiles.

In this part, participants with use of lipid-lowering drugs were excluded. This sensitive analysis was carried out within Model 5. Similar with previous outcomes, the results still showed an apparently increasing risk of hyperuricemia with across the quantiles of AIP, which enhanced the robustness (Figure 5).

## 4. Discussion

This nationwide study estimated that approximately 5.2% of the middle-aged and above Chinese adults suffered from hyperuricemia in 2011, while the prevalence increased to 9.5% in the target population in 2015. AIP was demonstrated positively associated with the prevalence of hyperuricemia in the middle-aged and elderly Chinese. Though there was no nonlinear relationship found, the curve of restricted cubic splines analysis generally exhibited a positive relationship between AIP and hyperuricemia, indicating AIP has a proper predictive ability for hyperuricemia. This positive association still existed after the adjustment for confounding factors by PSM.

Hyperuricemia has gradually become an important public health issue worldwide [30]. Especially in China, an alarming rise was observed in recent years attributed to its growing economy and urbanization [6], which is in accordance with the findings of this paper. It is worth noting that the prevalence of hyperuricemia varies in different geographic regions in China. This phenomenon might be related to variability in economic development, resulting in the lifestyle difference [5]. A huge dietary transition occurred from relying on carbohydrates and vegetables to meat and dairy products, in which bacon, beef, lamb, and organ meats were purine-rich diet and identified as the main pathogenic factors of hyperuricemia [31]. Besides, alcohol was one dependent risk factor for hyperuricemia [32]. Similarly, in our study, an apparent elevated risk for hyperuricemia was found in the participants drinking regularly than those drinking occasionally or never drink. The risk of dyslipidemia increased due to the diet high in fat and protein [33].

Previous findings also documented that hyperuricemia is positively associated with dyslipidemia in northwest China [28]. Besides, a cross-sectional study in Chinese at 2020 revealed HDL-C is a protective predictor of serum uric acid levels in gout [34]. Similarly, a significant negative correlation was observed between uric acid level and HDL-C among young adults in Qatar [35]. In addition, a large retrospective cohort study revealed that TG levels independently affect the incidence of hyperuricemia [36]. The mechanism behind the increasing AIP and elevated TG levels is not fully clear. The possible mechanism may be mainly related to the following points. First, TG links with high uric acid may involve free fatty acids metabolic pathways, which is a physiological process for the long-term regulation of energy homeostasis [37]. TG can take part in the synthesis of free fatty acids, in which the utilization of adenosine triphosphate in the process of re-esterification was accelerated. Elevate TG can lead to a high level of free fatty acid production and utilization, which could accelerate the decomposition of adenosine triphosphate, and this process increased the production of uric acid and causes its abnormal rise in blood [38]. Second, many studies have confirmed that HDL-C is a protective factor against serum uric acid elevation in general [39–41].

Besides, a major protein component of HDL-C called apolipoprotein A-1 exerts an inhibitory effect on monocytes cytokines by reducing the activation of CD11b, since the monocytes can elevate serum uric acid levels through different pathways [42, 43]. Thus, it makes sense that AIP, in which TG serves as a numerator while HDL acts as a denominator, is positively correlated with hyperuricemia.

Even so, AIP was rarely studied in hyperuricemia. Duan et al. have proved hyperuricemia is an independent risk factor for high AIP levels a cross-sectional study at 2022 [44]. But it made little sense to utilize a disease to predict an indicator. In the cross-sectional analysis part of our study, every single model demonstrated an apparently positive association between AIP and the prevalence of hyperuricemia. Especially in the fully adjusted model, the risk of hyperuricemia in the highest AIP quartile is 2.81 times bigger than the reference group. Further, RCS analysis also shows an upward trend of hyperuricemia with the AIP increasing. In addition, after attenuating the effect of confounding factors by PSM, both the univariate and multivariate logistic models were still robust; the risk of hyperuricemia increased 1.59 and 1.37 times, respectively. Besides, this trend still held in the sensitive analysis for the use of lipid-lowering drugs. A cross-Sectional study from northeast China suggested that AIP can predict hyperuricemia in rural population of northeast China [45], which was consistent with our findings; besides, it showed that, compared to the low AIP group (<0.11), participants in increased AIP group (>0.21) had a 2.536-fold risk for hyperuricemia. In our study, after excluding the participants with hyperuricemia in 2011, the longitudinal analysis was conducted between CHARLS 2015 and 2011 survey. Similar outcomes showed that the risk of hyperuricemia apparently increased with the growth of AIP in all models compared with the reference group. Even after PSM, the outcome maintained accordantly. In addition, this longitudinal analysis research was based on a national scale survey of CHARLS, suggesting that AIP has the ability to predict the prevalence of hyperuricemia in the middle-aged and elderly Chinese population.

Generally, we found a practical indicator for predicting hyperuricemia prevalence, and it enriched the method alarming the hyperuricemia attack. In addition, AIP is a low-cost indicator because both TG and HDL are the basic items in the routine blood biochemical inspection on hospital admission or medical examination, which cost little to carry out. Besides, it is a feasible indicator to acquire, for it can be directly calculated and does not need more complicated physical examination and inspection. Given this, AIP is a high-quality and cost-effective indicator to alarm the occurrence of hyperuricemia, which has important clinical implications and may be a promising indicator in clinical practice. Nevertheless, further exploration is still demanded. To the best of our knowledge, this is the first report to investigate the longitudinal relationship between AIP and hyperuricemia in the middle-aged and elderly Chinese population.

Our study also had several limitations. First, this is a retrospective study, which is limited to establish a causal relation between AIP and hyperuricemia. Therefore, large-scale prospective studies are needed to verify the present findings. Second, hyperuricemia is a disease interacted closely with diet, while the diet documented in CHARLS is not that sufficient, and this should be improved with the completeness of the database. Third, the information collected from the participants may suffer from recall bias on self-report variables, so it still has chance to cause deviation. Finally, this study was based on the CHARLS survey in 2011 and 2015, which means it should be further verified in other countries or regions worldwide.

## 5. Conclusion

Conclusively, this pilot study suggested that AIP can predict the prevalence of hyperuricemia in Chinese middle-aged and elderly population.

## Figures and Tables

**Table 1:** Baseline clinical profiles of participants attending the 2011 CHARLS survey.

Clinical characteristics	Total participants	*Q*1	*Q*2	*Q*3	*Q*4	*P*
	*N* = 13,021	*N* = 3,275	*N* = 3,236	*N* = 3,246	*N* = 3,264	
Age, mean (SD)	59.21 (9.71)	59.83 (10.10)	59.38 (9.91)	59.11 (9.52)	58.51 (9.26)	<0.001
Gender						<0.001
Male	5367 (46.6%)	1497 (51.9%)	1320 (46.0%)	1258 (43.6%)	1292 (45.0%)	
Female	6146 (53.4%)	1390 (48.1%)	1550 (54.0%)	1625 (56.4%)	1581 (55.0%)	
Marital status						0.003
With spouse present	9528 (82.8%)	2374 (82.2%)	2354 (82.0%)	2358 (81.8%)	2442 (85.0%)	
Others	1985 (17.2%)	513 (17.8%)	516 (18.0%)	525 (18.2%)	431 (15.0%)	
Cigarette consumption						<0.001
Current smoker	3512 (30.5%)	982 (34.0%)	886 (30.9%)	830 (28.8%)	814 (28.3%)	
Nonsmoker	7026 (61.1%)	1660 (57.5%)	1760 (61.4%)	1823 (63.3%)	1783 (62.1%)	
Ever-smoker	970 (8.4%)	244 (8.5%)	222 (7.7%)	229 (7.9%)	275 (9.6%)	
Alcohol consumption						<0.001
Drink more than once a month	2870 (24.9%)	935 (32.4%)	670 (23.4%)	620 (21.5%)	645 (22.5%)	
Drink less than once a month	900 (7.8%)	227 (7.9%)	229 (8.0%)	213 (7.4%)	231 (8.0%)	
None of these	7736 (67.2%)	1724 (59.7%)	1969 (68.7%)	2048 (71.1%)	1995 (69.5%)	
Body mass index						<0.001
<18.5 kg/m^2^	669 (6.9%)	290 (11.7%)	215 (8.8%)	121 (5.0%)	43 (1.8%)	
≥18.5 & <24 kg/m^2^	5035 (51.9%)	1661 (66.8%)	1373 (56.3%)	1120 (46.4%)	881 (37.3%)	
≥24 & <28 kg/m^2^	2851 (29.4%)	438 (17.6%)	652 (26.7%)	824 (34.1%)	937 (39.7%)	
≥28 kg/m^2^	1148 (11.8%)	99 (4.0%)	199 (8.2%)	348 (14.4%)	502 (21.2%)	
Nap						0.004
No	5154 (46.7%)	1347 (48.5%)	1300 (47.2%)	1298 (47.2%)	1209 (43.8%)	
Yes	5889 (53.3%)	1432 (51.5%)	1455 (52.8%)	1451 (52.8%)	1551 (56.2%)	
Sleep duration						0.30
<6 hours	5548 (50.6%)	1422 (51.4%)	1386 (50.7%)	1405 (51.6%)	1335 (48.7%)	
6–8 hours	4486 (40.9%)	1118 (40.4%)	1105 (40.4%)	1088 (40.0%)	1175 (42.9%)	
>8 hours	928 (8.5%)	226 (8.2%)	243 (8.9%)	229 (8.4%)	230 (8.4%)	
Low-density lipoprotein						<0.001
<120 mg/dL	6586 (57.3%)	1894 (65.6%)	1551 (54.0%)	1396 (48.4%)	1745 (61.2%)	
≥120 mg/dL	4905 (42.7%)	992 (34.4%)	1319 (46.0%)	1486 (51.6%)	1108 (38.8%)	
Total cholesterol						<0.001
<200 mg/dL	6991 (60.7%)	1934 (67.0%)	1850 (64.5%)	1711 (59.4%)	1496 (52.1%)	
≥200 mg/dL	4519 (39.3%)	952 (33.0%)	1019 (35.5%)	1171 (40.6%)	1377 (47.9%)	
Depression	3980 (38.1%)	1039 (39.7%)	982 (37.8%)	1003 (38.5%)	956 (36.5%)	0.12
Arthritis	4032 (35.0%)	979 (33.9%)	1004 (35.0%)	1057 (36.7%)	992 (34.5%)	0.15
Digestive diseases	2659 (23.1%)	729 (25.3%)	679 (23.7%)	650 (22.5%)	601 (20.9%)	0.001
Renal diseases	772 (6.7%)	205 (7.1%)	184 (6.4%)	188 (6.5%)	195 (6.8%)	0.73
Hepatic diseases	451 (3.9%)	117 (4.1%)	101 (3.5%)	117 (4.1%)	116 (4.0%)	0.66

*N*: number; *Q*: quartile; CHARLS: China Health and Retirement Longitudinal Study.

**Table 2:** Odds ratios (95% CI) for hyperuricemia by quartiles of AIP.

Quartiles of AIP	*Q*1	*Q*2	*Q*3	*Q*4	*P* for trend
Cases	94	131	143	303	—
Prevalence (%)	3.26	4.56	4.96	10.55	—
Model 1	1.00 (reference)	1.42 (1.08–1.86)^*∗*^	1.55 (1.19–2.02)^*∗∗∗*^	3.50 (2.76–4.44)^*∗∗∗*^	<0.001
Model 2	1.00 (reference)	1.52 (1.15–2.02)^*∗∗*^	1.79 (1.36–2.36)^*∗∗∗*^	4.18 (3.26–5.36)^*∗∗∗*^	<0.001
Model 3	1.00 (reference)	1.47 (1.07–2.01)	1.73 (1.27–2.36)^*∗∗∗*^	3.45 (2.60–4.65)^*∗∗∗*^	<0.001
Model 4	1.00 (reference)	1.52 (1.11–2.10)^*∗∗*^	1.82 (1.32–2.49)^*∗∗∗*^	3.81 (2.84–5.11)^*∗∗∗*^	<0.001
Model 5	1.00 (reference)	1.52 (1.10–2.10)^*∗∗*^	1.80 (1.31–2.47)^*∗∗∗*^	3.81 (2.84–5.11)^*∗∗∗*^	<0.001

Logistic regression to examine the association between AIP index and hyperuricemia. *Q*1 was set as the reference group. Model 1: univariate regression; Model 2: adjusted by age, gender, marital status, sleeping time, and afternoon napping based on model 1; Model 3: further adjusted by alcohol and cigarette consumption, BMI and depression; Model 4: further adjusted by blood uric acid and LDL; Model 4: further adjusted by hepatic disease, digestive disease, renal disease, and arthritis disease. ^*∗*^*p* < 0.05; ^*∗∗*^*p* < 0.01; ^*∗∗∗*^*p* < 0.001.

**Table 3:** Subgroup analysis of association between quartiles of AIP and hyperuricemia.

Factors	*Q*1	*Q*2	*Q*3	*Q*4	*P* for interaction
Gender					0.80
Male	1.00	1.41 (0.92–2.16)	1.82 (1.20–2.77)^*∗∗*^	3.78 (2.55–5.62)^*∗∗∗*^	
Female	1.00	1.60 (0.97–2.62)	1.68 (1.03–2.75)^*∗*^	3.07 (1.3–4.90)^*∗∗∗*^	
Renal disease					0.16
Yes	1.00	2.04 (0.55–7.54)	5.38 (1.61–17.87)^*∗∗*^	4.86 (1.44–16.35)	
No	1.00	1.50 (1.08–2.09)^*∗*^	1.63 (1.17–2.27)^*∗∗*^	3.40 (2.50–4.64)^*∗∗∗*^	
Alcohol consumption					0.02
More than once a month	1.00	1.72 (0.97–3.03)	1.92 (1.08–3.39)^*∗*^	4.71 (2.79–7.94)^*∗∗∗*^	
Less than once a month	1.00	1.08 (0.29–4.06)	0.99 (0.24–4.00)	2.15 (0.62–7.38)	
Nondrinker	1.00	1.40 (0.93–2.12)	1.66 (1.11–2.48)^*∗*^	2.94 (2.00–4.31)^*∗∗∗*^	
Cigarette consumption					0.53
Current smoker	1.00	1.24 (0.72–2.12)	1.35 (0.78–2.33)	2.64 (1.58–4.41)^*∗∗∗*^	
Nonsmoker	1.00	1.81 (1.16–2.82)	1.97 (1.27–3.06)^*∗∗*^	3.51 (2.30–5.35)^*∗∗∗*^	
Ever-smoker	1.00	0.59 (0.22–1.58)	1.06 (0.44–2.55)	3.21 (1.47–7.00)^*∗∗*^	
Depression					0.78
Yes	1.00	1.64 (0.95–2.84)	2.29 (1.36–3.87)^*∗∗*^	3.57 (2.14–5.95)^*∗∗∗*^	
No	1.00	1.44 (0.97–2.15)	1.51 (1.00–2.25)^*∗*^	3.40 (2.36–4.92)^*∗∗∗*^	
Sleep duration					0.97
<6 hours	1.00	1.92 (1.26–2.92)^*∗∗*^	1.82 (1.19–2.81)^*∗∗*^	3.29 (2.19–4.95)^*∗∗∗*^	
6–8 hours	1.00	1.31 (0.76–2.26)	1.72 (1.02–2.91)^*∗*^	3.73 (2.30–6.06)^*∗∗∗*^	
>8 hours	1.00	0.40 (0.08–2.12)	1.96 (0.62–6.19)	3.87 (1.23–12.18)^*∗*^	

Association between quartiles of AIP and hyperuricemia stratified by gender, renal disease, alcohol consumption, cigarette consumption, depression, and sleep duration. *Q*1 was set as the reference group. Models were adjusted for the same covariates as model 5 in Table 2. Stratification variables were not adjusted in the corresponding models. *Q*: quartile; ^*∗*^*p* < 0.05; ^*∗∗*^*p* < 0.01; ^*∗∗∗*^*p* < 0.001.

**Table 4:** Odds ratio (95% CI) for prevalent hyperuricemia by quartiles of AIP in longitudinal analysis.

Quartiles of AIP	*Q*1	*Q*2	*Q*3	*Q*4	*P* for trend
Cases	107	114	185	258	—
Prevalence (%)	5.92	6.48	10.37	15.48	—
Model 1	1.00 (reference)	1.10 (0.84–1.45)	1.84 (1.44–2.36)^*∗∗∗*^	2.91 (2.30–3.69)^*∗∗∗*^	<0.001
Model 2	1.00 (reference)	1.17 (0.86–1.55)	1.95 (1.51–2.52)^*∗∗∗*^	3.21 (2.52–4.10)^*∗∗∗*^	<0.001
Model 3	1.00 (reference)	1.04 (0.77–1.40)	1.65 (1.25–2.17)^*∗∗∗*^	2.58 (1.97–3.38)^*∗∗∗*^	<0.001
Model 4	1.00 (reference)	1.01 (0.75–1.37)	1.59 (1.20–2.10)^*∗∗∗*^	2.53 (1.93–3.33)^*∗∗∗*^	<0.001
Model 5	1.00 (reference)	1.00 (0.74–1.37)	1.59 (1.20–2.11)^*∗∗∗*^	2.55 (1.94–3.35)^*∗∗∗*^	<0.001

Odds ratio (95% CI) for prevalent hyperuricemia by quartiles of AIP in longitudinal analysis. The adjusting variables were the same as Table 2. ^*∗*^*p*  <  0.05; ^*∗∗*^*p*  <  0.01; ^*∗∗∗*^*p*  <  0.001.

**Table 5:** Subgroups analysis of association between quartiles of AIP and hyperuricemia in longitudinal analysis.

Factors	*Q*1	*Q*2	*Q*3	*Q*4	*P* for interaction
Gender					0.09
Male	1.00	1.20 (0.80–1.80)	1.67 (1.13–2.49)^*∗*^	2.32 (1.57–3.46)^*∗∗∗*^	
Female	1.00	0.81 (0.51–1.30)	1.53 (1.01–2.30)^*∗*^	2.63 (1.77–3.92)^*∗∗∗*^	
Renal disease					0.06
Yes	1.00	0.72 (0.17–3.16)	2.81 (0.77–10.25)	7.60 (2.37–24.38)^*∗∗∗*^	
No	1.00	1.02 (0.75–1.40)	1.53 (1.14–2.01)^*∗∗*^	2.26 (1.69–3.01)^*∗∗∗*^	
Alcohol consumption					0.09
More than once a month	1.00	1.11 (0.66–1.87)	1.52 (0.91–2.54)	2.20 (1.31–3.70)^*∗∗*^	
Less than once a month	1.00	1.16 (0.43–3.15)	0.98 (0.33–2.91)	2.79 (1.07–7.23)^*∗*^	
Nondrinker	1.00	0.95 (0.63–1.44)	1.74 (1.20–2.52)^*∗∗*^	2.65 (1.84–3.82)^*∗∗∗*^	
Cigarette consumption					0.02
Current smoker	1.00	1.04 (0.64–1.71)	1.15 (0.70–1.89)	1.69 (1.04–2.75)^*∗*^	
Nonsmoker	1.00	1.01 (0.67–1.52)	1.78 (1.22–2.60)^*∗∗*^	2.74 (1.90–3.95)^*∗∗∗*^	
Ever-smoker	1.00	0.73 (0.18–2.90)	2.85 (0.93–8.76)	5.49 (1.84–16.41)^*∗∗*^	
Depression					0.88
Yes	1.00	1.12 (0.69–1.83)	1.35 (0.85–2.15)	2.38 (1.51–3.74)^*∗∗∗*^	
No	1.00	0.96 (0.65–1.43)	1.75 (1.22–2.62)^*∗∗*^	2.61 (1.83–3.72)^*∗∗∗*^	
Sleep duration					0.87
<6 hours	1.00	0.97 (0.62–1.51)	1.65 (1.11–2.47)^*∗∗*^	2.76 (1.86–4.09)^*∗∗∗*^	
6–8 hours	1.00	0.93 (0.58–1.50)	1.40 (0.90–2.17)	2.15 (1.41–3.27)^*∗∗∗*^	
>8 hours	1.00	1.83 (0.61–5.49)	2.51 (0.88–7.18)	2.90 (0.97–8.65)	

Subgroup analysis of association between quartiles of AIP and hyperuricemia. Models were adjusted for the same covariates as model 5 in Table 2. *Q*1 was set as the reference group. Stratification variables were not adjusted in the corresponding models. *Q*: quartile; ^*∗*^*p* < 0.05; ^*∗∗*^*p* < 0.01; ^*∗∗∗*^*p* < 0.001.

## Data Availability

The datasets generated and/or analyzed during the current study are available in the CHARLS repository https://charls.pku.edu.cn/.

## Supplementary Materials

Supplementary Materials Supplementary Figure 1 and Supplementary Figure 2 displayed the outcomes of PSM before cross-sectional analysis and before longitudinal analysis, respectively. All the standardized bias across covariates was less than 10%, which means the PSM was performed successfully. The Supplementary Table 1 and Supplementary Table 2 showed the baseline characteristics after PSM; most of the covariates showed no statistical significance, indicating the confounder effect were attenuated to a large extent.

## References

[1] George C., Minter D. A. (2023). Hyperuricemia. *StatPearls. 2023, StatPearls Publishing Copyright ©*.

[2] Fathallah-Shaykh S. A., Cramer M. T. (2014). Uric acid and the kidney. *Pediatric Nephrology*.

[3] Trifirò G., Morabito P., Cavagna L. (2013). Epidemiology of gout and hyperuricaemia in Italy during the years 2005-2009: a nationwide population-based study. *Annals of the Rheumatic Diseases*.

[4] Zhu Y., Pandya B. J., Choi H. K. (2011). Prevalence of gout and hyperuricemia in the US general population: the national health and nutrition examination survey 2007-2008. *Arthritis and Rheumatism*.

[5] Liu R., Han C., Wu D. (2015). Prevalence of hyperuricemia and gout in mainland China from 2000 to 2014: a systematic review and meta-analysis. *BioMedical Research International*.

[6] Zhang M., Zhu X., Wu J. (2021). Prevalence of hyperuricemia among Chinese adults: findings from two nationally representative cross-sectional surveys in 2015-16 and 2018-19. *Frontiers in Immunology*.

[7] Waheed Y., Yang F., Sun D. (2021). Role of asymptomatic hyperuricemia in the progression of chronic kidney disease and cardiovascular disease. *Korean Journal of Internal Medicine (English Edition)*.

[8] Shahin L., Patel K. M., Heydari M. K., Kesselman M. M. (2021). Hyperuricemia and cardiovascular risk. *Cureus*.

[9] Eun Y., Han K. D., Kim D. H. (2020). Increased overall heart rate irregularity risk by hyperuricemia in the general population: results from the Korean national health and nutrition examination survey. *Medicina*.

[10] Zuo T., Liu X., Jiang L., Mao S., Yin X., Guo L. (2016). Hyperuricemia and coronary heart disease mortality: a meta-analysis of prospective cohort studies. *BioMedical Central Cardiovascular Disorders*.

[11] Padda J., Khalid K., Almanie A. H. (2021). Hyperuricemia in patients with coronary artery disease and its association with disease severity. *Cureus*.

[12] Holme I., Aastveit A. H., Hammar N., Jungner I., Walldius G. (2009). Uric acid and risk of myocardial infarction, stroke and congestive heart failure in 417,734 men and women in the Apolipoprotein MOrtality RISk study (AMORIS). *Journal of Internal Medicine*.

[13] Li J., Muraki I., Imano H. (2020). Serum uric acid and risk of stroke and its types: the Circulatory Risk in Communities Study (CIRCS). *Hypertension Research*.

[14] Zhang S., Wang Y., Cheng J. (2019). Hyperuricemia and cardiovascular disease. *Current Pharmaceutical Design*.

[15] Karantas I. D., Okur M. E., Okur N. Ü, Siafaka P. I. (2021). Dyslipidemia management in 2020: an update on diagnosis and therapeutic perspectives. *Endocrine, Metabolic & Immune Disorders-Drug Targets*.

[16] Santangelo G., Moscardelli S., Simeoli P. S., Guazzi M., Faggiano P. (2022). Management of dyslipidemia in secondary prevention of cardiovascular disease: the gap between theory and practice. *Journal of Clinical Medicine*.

[17] Ference B. A., Ginsberg H. N., Graham I. (2017). Low-density lipoproteins cause atherosclerotic cardiovascular disease. 1. Evidence from genetic, epidemiologic, and clinical studies. A consensus statement from the European Atherosclerosis Society Consensus Panel. *European Heart Journal*.

[18] Peng T. C., Wang C. C., Kao T. W. (2015). Relationship between hyperuricemia and lipid profiles in US adults. *BioMedical Research International*.

[19] Dobiás̆ová M., Frohlich J. (2001). The plasma parameter log (TG/HDL-C) as an atherogenic index: correlation with lipoprotein particle size and esterification rate in apoB-lipoprotein-depleted plasma (FER(HDL)). *Clinical Biochemistry*.

[20] Nam J. S., Kim M. K., Nam J. Y. (2020). Association between atherogenic index of plasma and coronary artery calcification progression in Korean adults. *Lipids in Health and Disease*.

[21] Si Y., Fan W., Han C., Liu J., Sun L. (2021). Atherogenic index of plasma, triglyceride-glucose index and monocyte-to-lymphocyte ratio for predicting subclinical coronary artery disease. *The American Journal of the Medical Sciences*.

[22] Hartopo A. B., Arso I. A., Setianto B. Y. (2016). Low plasma atherogenic index associated with poor prognosis in hospitalized patients with acute myocardial infarction. *Acta Medical Indones*.

[23] Zhao Y., Hu Y., Smith J. P., Strauss J., Yang G. (2014). Cohort profile: the China health and retirement longitudinal study (CHARLS). *International Journal of Epidemiology*.

[24] Andresen E. M. (2013). Performance of the 10-item center for epidemiologic studies depression scale for caregiving research. *Sage Open Medicine*.

[25] Zhu X. X., Dai Y. X. (2017). Chinese multi-disciplinary consensus on the diagnosis and treatment of hyperuricemia and its related diseases. *Zhonghua Nei Ke Za Zhi*.

[26] Gao Y., Cui L., Sun Y. (2021). Adherence to the dietary approaches to stop hypertension diet and hyperuricemia: a cross-sectional study. *Arthritis Care and Research*.

[27] Song P., Wang H., Xia W., Chang X., Wang M., An L. (2018). Prevalence and correlates of hyperuricemia in the middle-aged and older adults in China. *Scientific Reports*.

[28] Liu F., Du G. L., Song N. (2020). Hyperuricemia and its association with adiposity and dyslipidemia in Northwest China: results from cardiovascular risk survey in Xinjiang (CRS 2008-2012). *Lipids in Health and Disease*.

[29] Yuan J. H., Xiong Y., Zhang Y. C., Jin T., Qin F. (2021). Depressive males have higher odds of lower urinary tract symptoms suggestive of benign prostatic hyperplasia: a retrospective cohort study based on propensity score matching. *Asian Journal of Andrology*.

[30] Dehlin M., Jacobsson L., Roddy E. (2020). Global epidemiology of gout: prevalence, incidence, treatment patterns and risk factors. *Nature Reviews Rheumatology*.

[31] Yokose C., McCormick N., Choi H. K. (2021). Dietary and lifestyle-centered approach in gout care and prevention. *Current Rheumatology Reports*.

[32] He H., Pan L., Ren X. (2022). The effect of body weight and alcohol consumption on hyperuricemia and their population attributable fractions: a national health survey in China. *Obesity Facts*.

[33] Trautwein E. A., McKay S. (2020). The role of specific components of a plant-based diet in management of dyslipidemia and the impact on cardiovascular risk. *Nutrients*.

[34] Liang J., Jiang Y., Huang Y. (2020). The comparison of dyslipidemia and serum uric acid in patients with gout and asymptomatic hyperuricemia: a cross-sectional study. *Lipids in Health and Disease*.

[35] Al Shanableh Y., Hussein Y. Y., Saidwali A. H. (2022). Prevalence of asymptomatic hyperuricemia and its association with prediabetes, dyslipidemia and subclinical inflammation markers among young healthy adults in Qatar. *BioMedical Central Endocrine Disorders*.

[36] Zhang L., Wan Q., Zhou Y. (2019). Age-related and gender-stratified differences in the association between high triglyceride and risk of hyperuricemia. *Lipids in Health and Disease*.

[37] Balasubramanian T. (2003). Uric acid or 1-methyl uric acid in the urinary bladder increases serum glucose, insulin, true triglyceride, and total cholesterol levels in Wistar rats. *The Scientific World Journal*.

[38] Zhang Y., Wei F., Chen C. (2018). Higher triglyceride level predicts hyperuricemia: a prospective study of 6-year follow-up. *Journal of Clinical Lipidology*.

[39] Zou X., Zhao Z., Huang W. (2023). High-density lipoprotein cholesterol modifies the association between blood lead and uric acid: results from NHANES 2005-2016. *International Archives of Occupational and Environmental Health*.

[40] Li Y., Liu X., Luo Y. (2022). Monocyte to high-density lipoprotein cholesterol ratio and serum uric acid in Chinese adults: a cross-sectional study. *BioMedical Central Endocrine Disorders*.

[41] Murphy A. J., Woollard K. J. (2010). High-density lipoprotein: a potent inhibitor of inflammation. *Clinical and Experimental Pharmacology and Physiology*.

[42] Murphy A. J., Woollard K. J., Hoang A. (2008). High-density lipoprotein reduces the human monocyte inflammatory response. *Arteriosclerosis, Thrombosis, and Vascular Biology*.

[43] Varrin-Doyer M., Spencer C. M., Schulze‐Topphoff U., Nelson P. A., Stroud R. A., Zamvil S. S. (2012). Aquaporin 4-specific T cells in neuromyelitis optica exhibit a Th17 bias and recognize Clostridium ABC transporter. *Annals of Neurology*.

[44] Duan Y., Chang X., Ding X., An Y., Wang G., Liu J. (2022). Association of hyperuricemia with apolipoprotein AI and atherogenic index of plasma in healthy Chinese people: a cross-sectional study. *BioMedical Central Cardiovascular Disorders*.

[45] Chang Y., Li Y., Guo X., Guo L., Sun Y. (2016). Atherogenic index of plasma predicts hyperuricemia in rural population: a cross-sectional study from northeast China. *International Journal of Environmental Research and Public Health*.

[46] Xu F., Ma C., Wang S. (2023). *Higher Atherogenic index of Plasma Is Associated with Hyperuricemia: A National Longitudinal Study*.

