# Supplementary material for: Higher Atherogenic Index of Plasma Is Associated with Hyperuricemia: A National Longitudinal Study
Source: Int J Endocrinol. 2024 Feb 19;2024:4002839. doi: 10.1155/2024/4002839 (PMC10896650; doi:10.1155/2024/4002839)
Supplement: Supplementary Materials — Supplementary Figure 1 and Supplementary Figure 2 displayed the outcomes of PSM before cross-sectional analysis and before longitudinal analysis, respectively. All the standardized bias across covariates was less than 10%, which means the PSM was performed successfully. The Supplementary Table 1 and Supplementary Table 2 showed the baseline characteristics after PSM; most of the covariates showed no statistical significance, indicating the confounder effect were attenuated to a large extent. [file 4002839.f1.zip › supplemental figures.pdf]

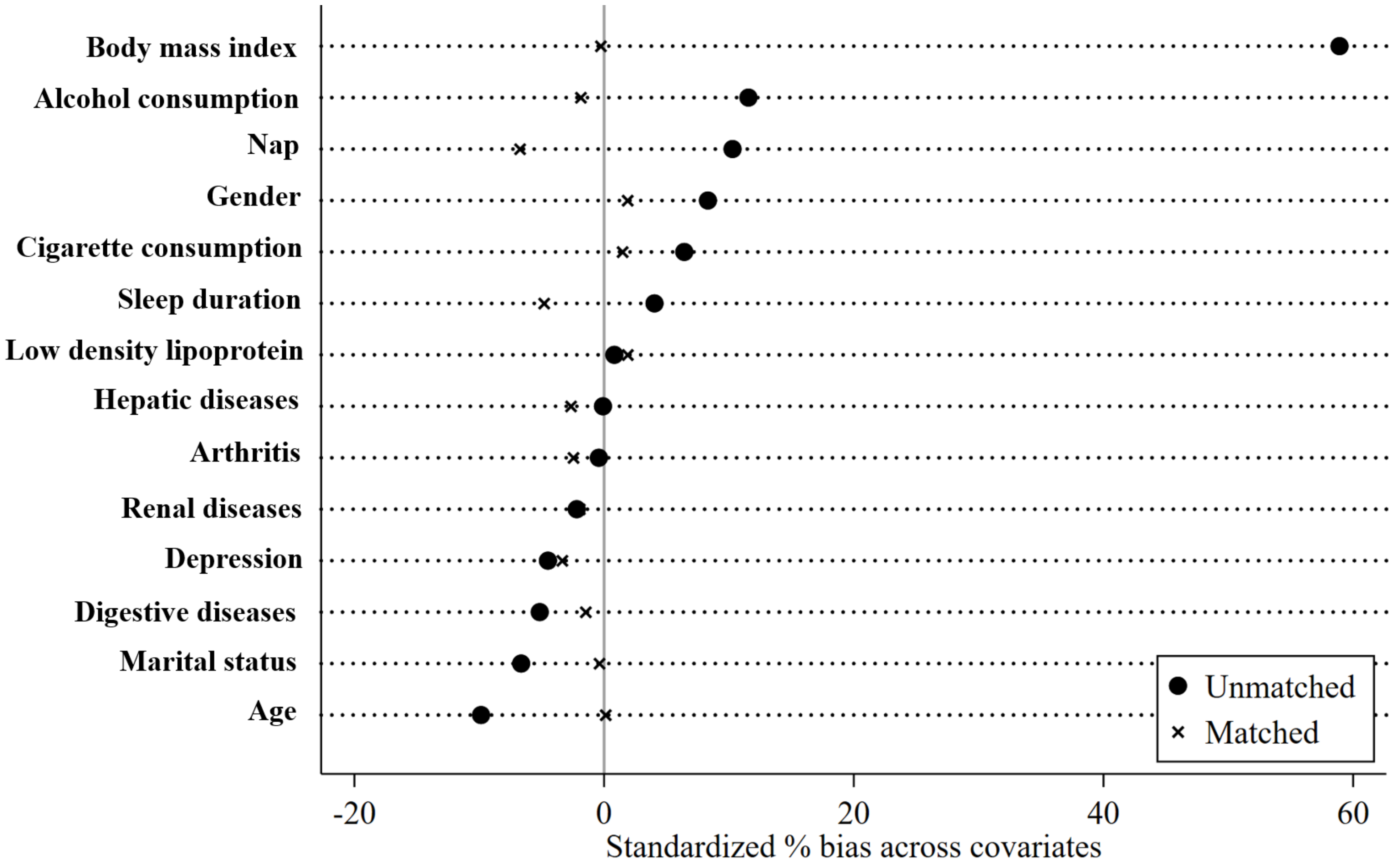

Supplemental figure1 Standardized bias across covariates in the cross-sectional analysis All the standardized bias was less than 10%.

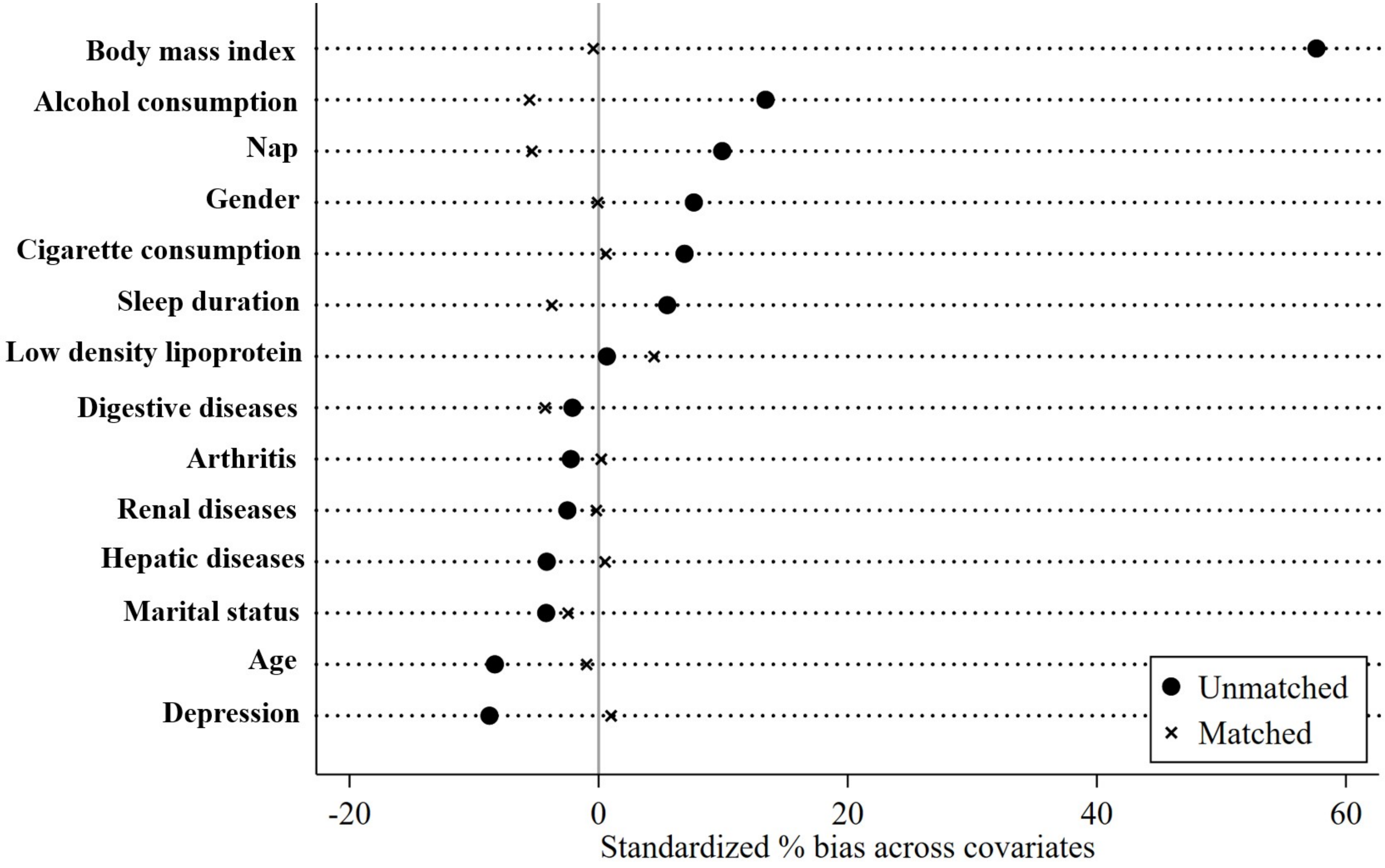

Supplemental figure2 Standardized bias across covariates in the longitudinal analysis All the standardized bias was less than 10%.
