# Supplementary material for: Higher Atherogenic Index of Plasma Is Associated with Hyperuricemia: A National Longitudinal Study
Source: Int J Endocrinol. 2024 Feb 19;2024:4002839. doi: 10.1155/2024/4002839 (PMC10896650; doi:10.1155/2024/4002839)
Supplement: Supplementary Materials — Supplementary Figure 1 and Supplementary Figure 2 displayed the outcomes of PSM before cross-sectional analysis and before longitudinal analysis, respectively. All the standardized bias across covariates was less than 10%, which means the PSM was performed successfully. The Supplementary Table 1 and Supplementary Table 2 showed the baseline characteristics after PSM; most of the covariates showed no statistical significance, indicating the confounder effect were attenuated to a large extent. [file 4002839.f1.zip › supplemental tables.docx]

**Supplemental table 1** **The baseline characteristics after PSM for cross-sectional analysis**

| **Clinical characteristics** | **Participants** | **AIP<1.05** | **AIP≥1.05** | **P** |
| --- | --- | --- | --- | --- |
|  | **N = 5,800** | **N = 2,900** | **N = 2,900** |  |
| **Age, mean (SD)** | 58.62 (9.16) | 58.64 (9.30) | 58.61 (9.02) | 0.90 |
| **Gender** |  |  |  | 0.89 |
| **Male** | 2559 (44.1%) | 1282 (44.2%) | 1277 (44.0%) |  |
| **Female** | 3241 (55.9%) | 1618 (55.8%) | 1623 (56.0%) |  |
| **Marital status** |  |  |  | 0.66 |
| **With spouse present** | 4898 (84.4%) | 2443 (84.2%) | 2455 (84.7%) |  |
| **Others** | 902 (15.6%) | 457 (15.8%) | 445 (15.3%) |  |
| **Cigarette consumption** |  |  |  | 0.04 |
| **Current-smoker** | 1635 (28.2%) | 797 (27.5%) | 838 (28.9%) |  |
| **Non-smoker** | 3640 (62.8%) | 1862 (64.2%) | 1778 (61.3%) |  |
| **Ever-smoker** | 525 (9.1%) | 241 (8.3%) | 284 (9.8%) |  |
| **Alcohol consumption** |  |  |  | 0.60 |
| **Drink more than once a month** | 1323 (22.8%) | 653 (22.5%) | 670 (23.1%) |  |
| **Drink less than once a month** | 472 (8.1%) | 228 (7.9%) | 244 (8.4%) |  |
| **None of These** | 4005 (69.1%) | 2019 (69.6%) | 1986 (68.5%) |  |
| **Body mass index** | 86.50 (12.57) | 85.52 (12.60) | 87.47 (12.47) | <0.001 |
| **< 18.5 Kg/m2** |  |  |  | 0.67 |
| **≥ 18.5 & < 24 Kg/m2** | 156 (2.7%) | 75 (2.6%) | 81 (2.8%) |  |
| **≥ 24 & < 28 Kg/m2** | 2463 (42.5%) | 1238 (42.7%) | 1225 (42.2%) |  |
| **≥ 28 Kg/m2** | 2345 (40.4%) | 1156 (39.9%) | 1189 (41.0%) |  |
| **Nap** | 836 (14.4%) | 431 (14.9%) | 405 (14.0%) |  |
| **No** |  |  |  | 0.03 |
| **Yes** | 2607 (44.9%) | 1261 (43.5%) | 1346 (46.4%) |  |
| **Sleep duration** | 3193 (55.1%) | 1639 (56.5%) | 1554 (53.6%) |  |
| **< 6 hours** |  |  |  | 0.57 |
| **6-8 hours** | 2907 (50.1%) | 1456 (50.2%) | 1451 (50.0%) |  |
| **> 8 hours** | 2425 (41.8%) | 1200 (41.4%) | 1225 (42.2%) |  |
| **Low density lipoprotein** | 468 (8.1%) | 244 (8.4%) | 224 (7.7%) |  |
| **< 120 mg/dL** |  |  |  | 0.77 |
| **≥ 120 mg/dL** | 3195 (55.1%) | 1603 (55.3%) | 1592 (54.9%) |  |
| **Total cholesterol** | 2605 (44.9%) | 1297 (44.7%) | 1308 (45.1%) |  |
| **< 200 mg/dL** |  |  |  | <0.001 |
| **≥ 200 mg/dL** | 3393 (58.5%) | 1854 (64.0%) | 1539 (53.1%) |  |
| **Depression** | 2406 (41.5%) | 1045 (36.0%) | 1361 (46.9%) |  |
| **Arthritis** | 2168 (37.4%) | 1089 (37.6%) | 1079 (37.2%) | 0.79 |
| **Digestive diseases** | 2065 (35.6%) | 1044 (36.0%) | 1021 (35.2%) | 0.53 |
| **Renal diseases** | 1345 (23.2%) | 676 (23.3%) | 669 (23.1%) | 0.83 |
| **Hepatic diseases** | 384 (6.6%) | 198 (6.8%) | 186 (6.4%) | 0.53 |

N: number; Q: quartile; CHARLS: China Health and Retirement Longitudinal Study

**Supplemental table 2 The baseline characteristics after PSM for longitudinal analysis**

| **Clinical characteristics** | **Participants** | **AIP<0.96** | **AIP≥0.96** | **P** |
| --- | --- | --- | --- | --- |
|  | **N = 4,192** | **N = 2,096** | **N = 2,096** |  |
| **Age, mean (SD)** | 58.36 (8.67) | 58.34 (8.74) | 58.38 (8.60) | 0.86 |
| **Gender** |  |  |  | 0.38 |
| **Male** | 1872 (44.7%) | 950 (45.3%) | 922 (44.0%) |  |
| **Female** | 2320 (55.3%) | 1146 (54.7%) | 1174 (56.0%) |  |
| **Marital status** |  |  |  | 0.79 |
| **With spouse present** | 3606 (86.0%) | 1806 (86.2%) | 1800 (85.9%) |  |
| **Others** | 586 (14.0%) | 290 (13.8%) | 296 (14.1%) |  |
| **Cigarette consumption** |  |  |  | 0.069 |
| **Current-smoker** | 1198 (28.6%) | 582 (27.8%) | 616 (29.4%) |  |
| **Non-smoker** | 2616 (62.4%) | 1341 (64.0%) | 1275 (60.8%) |  |
| **Ever-smoker** | 378 (9.0%) | 173 (8.3%) | 205 (9.8%) |  |
| **Alcohol consumption** |  |  |  | 0.82 |
| **Drink more than once a month** | 995 (23.7%) | 501 (23.9%) | 494 (23.6%) |  |
| **Drink less than once a month** | 347 (8.3%) | 168 (8.0%) | 179 (8.5%) |  |
| **None of These** | 2850 (68.0%) | 1427 (68.1%) | 1423 (67.9%) |  |
| **Body mass index** |  |  |  | 0.62 |
| **< 18.5 Kg/m2** | 94 (2.2%) | 45 (2.1%) | 49 (2.3%) |  |
| **≥ 18.5 & < 24 Kg/m2** | 1948 (46.5%) | 976 (46.6%) | 972 (46.4%) |  |
| **≥ 24 & < 28 Kg/m2** | 1637 (39.1%) | 806 (38.5%) | 831 (39.6%) |  |
| **≥ 28 Kg/m2** | 513 (12.2%) | 269 (12.8%) | 244 (11.6%) |  |
| **Nap** |  |  |  | 0.026 |
| **No** | 1920 (45.8%) | 924 (44.1%) | 996 (47.5%) |  |
| **Yes** | 2272 (54.2%) | 1172 (55.9%) | 1100 (52.5%) |  |
| **Sleep duration** |  |  |  | 0.62 |
| **< 6 hours** | 2122 (50.6%) | 1047 (50.0%) | 1075 (51.3%) |  |
| **6-8 hours** | 1744 (41.6%) | 880 (42.0%) | 864 (41.2%) |  |
| **> 8 hours** | 326 (7.8%) | 169 (8.1%) | 157 (7.5%) |  |
| **Low density lipoprotein** |  |  |  | 0.73 |
| **< 120 mg/dL** | 2311 (55.1%) | 1161 (55.4%) | 1150 (54.9%) |  |
| **≥ 120 mg/dL** | 1881 (44.9%) | 935 (44.6%) | 946 (45.1%) |  |
| **Total cholesterol** |  |  |  | <0.001 |
| **< 200 mg/dL** | 2480 (59.2%) | 1345 (64.2%) | 1135 (54.2%) |  |
| **≥ 200 mg/dL** | 1711 (40.8%) | 750 (35.8%) | 961 (45.8%) |  |
| **Depression** | 1583 (37.8%) | 785 (37.5%) | 798 (38.1%) | 0.68 |
| **Arthritis** | 1475 (35.2%) | 730 (34.8%) | 745 (35.5%) | 0.63 |
| **Digestive diseases** | 954 (22.8%) | 479 (22.9%) | 475 (22.7%) | 0.88 |
| **Renal diseases** | 259 (6.2%) | 125 (6.0%) | 134 (6.4%) | 0.56 |
| **Hepatic diseases** | 145 (3.5%) | 67 (3.2%) | 78 (3.7%) | 0.35 |

N: number; Q: quartile; CHARLS: China Health and Retirement Longitudinal Study
